# Investigation and management of acute hypoglycemia

**DOI:** 10.1186/1687-9856-2013-S1-O17

**Published:** 2013-10-03

**Authors:** Tohru Yorifuji

**Affiliations:** 1 Pediatric Endocrinology and Metabolism, Children’s Medical Center, Osaka City General Hospital, Osaka, Japan

## 

Since prolonged severe hypoglycemia could lead to permanent neurological sequelae, it must be treated immediately. But at the same time, the cause of hypoglycemia should be determined to prevent future recurrence of hypoglycemia preferably at bedside while the patient is still in an emergency room. Most of the cases, this could be done by asking proper questions and taking physical findings in parallel with the treatment, without waiting for the detailed results of critical samples. In taking history of the patients, one should not forget to ask the following questions; (1) preexisting medical problems (especially diabetes mellitus, CNS tumors, adrenal insufficiency, hepatic failure, arrhythmia, citrin deficiency or chronic infections), (2) medication history (especially insulin, oral hypoglycemic agents, beta-blocker, disopyramide, or extended use of antibiotics containing pivalic acid), (3) timing of hypoglycemia following the last meal (VERY important). In taking physical findings, presence or absence of hepatomegaly and circulatory collapse is the most important. Hypoglycemia within 2-3 hours after meal almost invariably suggests the presence of hyperinsulinemia. Hypoglycemia caused by a defect in glycogenolysis typically occurs 5-8 hours following the last meal and is usually accompanied by hepatomegaly. Recurrent episodes of hypoglycemia after an overnight fast likely suggest a problem in gluconeogenesis. Circulatory collapse at hypoglycemia suggests the presence of adrenal insufficiency. IV steroid in addition to IV glucose should be considered to prevent neurological sequelae. Critical samples should be taken at the time of hypoglycemia to make a causal diagnosis. Although the turnaround time of endocrine tests or tandem mass-spectrometry is usually long, results available on site often give a clue to the diagnosis. Relatively low ketone bodies by urine dipsticks suggest the presence of hyperinsulinemia or rare disorders of fatty-acid oxidation defects. Hyponatremia accompanied by hyperkalemia suggests primary (not secondary) adrenal insufficiency. Response to initial therapy also gives an important clue to the diagnosis. The need to continue IV glucose over normal resting hepatic glucose production to maintain normoglycemia (4-6 mg/kg^.^min in neonates) strongly suggests the presence of hyperinsulinemia. Never infuse too much glucose to the patients which could obscure the diagnosis. Finally, I will spend some time to show current state-of-the art in the diagnosis and management of congenital hyperinsulinism and discuss how the patients should be managed in the Asia-Pacific region.

